# Optimization of the production process for the anticancer lead compound illudin M: improving titers in shake-flasks

**DOI:** 10.1186/s12934-022-01827-z

**Published:** 2022-05-28

**Authors:** Lillibeth Chaverra-Muñoz, Theresa Briem, Stephan Hüttel

**Affiliations:** 1 Department of Microbial Drugs, Helmholtz Centre for Infection Research, Brunswick, Germany; 2 German Centre for Infection Research (DZIF), Partner Site Hannover-Braunschweig, Brunswick, Germany

**Keywords:** Natural products, Fungal secondary metabolites, Anti-cancer agents, Irofulven, Medium development, Bioprocess optimization, Small-scale, Shake-flask, Fed-batch, Acetate, Basidiomycota

## Abstract

**Background:**

The fungal sesquiterpenes Illudin M and S are important base molecules for the development of new anticancer agents due to their strong activity against some resistant tumor cell lines. Due to nonspecific toxicity of the natural compounds, improvement of the pharmacophore is required. A semisynthetic derivative of illudin S (Irofulven) entered phase II clinical trials for the treatment of castration-resistant metastatic prostate cancer. Several semisynthetic illudin M derivatives showed increased in vitro selectivity and improved therapeutic index against certain tumor cell lines, encouraging further investigation. This requires a sustainable supply of the natural compound, which is produced by Basidiomycota of the genus *Omphalotus*. We aimed to develop a robust biotechnological process to deliver illudin M in quantities sufficient to support medicinal chemistry studies and future preclinical and clinical development. In this study, we report the initial steps towards this goal.

**Results:**

After establishing analytical workflows, different culture media and commercially available *Omphalotus* strains were screened for the production of illudin M.*Omphalotus nidiformis* cultivated in a medium containing corn steep solids reached ~ 38 mg L^−1^ setting the starting point for optimization. Improved seed preparation in combination with a simplified medium (glucose 13.5 g L^−1^; corn steep solids 7.0 g L^− 1^; Dox broth modified 35 mL), reduced cultivation time and enhanced titers significantly (~ 400 mg L^−1^). Based on a reproducible cultivation method, a feeding strategy was developed considering potential biosynthetic bottlenecks. Acetate and glucose were fed at 96 h (8.0 g L^−1^) and 120 h (6.0 g L^−1^) respectively, which resulted in final illudin M titer of ~ 940 mg L^−1^ after eight days. This is a 25 fold increase compared to the initial titer.

**Conclusion:**

After strict standardization of seed-preparation and cultivation parameters, a combination of experimental design, empirical trials and additional supply of limiting biosynthetic precursors, led to a highly reproducible process in shake flasks with high titers of illudin M. These findings are the base for further work towards a scalable biotechnological process for a stable illudin M supply.

**Supplementary Information:**

The online version contains supplementary material available at 10.1186/s12934-022-01827-z.

## Background

Higher fungi have versatile life-cycles and complex metabolisms that enable adaption to many environmental conditions and thus they are important sources of interesting secondary metabolites such as polyketides, non-ribosomal peptides, terpenes and others [1, 2]. They are valuable producers of enzymes, organic acids, nutraceuticals, agrochemicals, and pharmaceuticals such as lefamulin, a pleuromutilin derivative recently launched for the treatment of community-acquired pneumonia [3–5]. Among fungi, Basidiomycota have been underexplored due to their complex biology, which hinders genetic manipulation and imposes difficulties for the application of genome mining tools which can be applied to other fungi [6]. Many of these fungi are difficult to cultivate under laboratory conditions, however they have been reported to produce diverse molecules with promising biological activities, and thus potential starting points for drug discovery programs if sufficient quantities of these molecules can be produced [7–9].

Interesting examples are the Illudins M and S, two potent cytotoxic sesquiterpenes discovered in the 1950’s by William J. Robbins and colleagues who isolated them from the culture broth of *Omphalotus illudens* formerly known as *Clitocybe illudens* or *Omphalotus olearius* [10, 11]. In the following decades illudins have been extensively studied because of their strong activity against tumor cell lines resistant to conventional anticancer drugs [12]. Illudin M and S are potent alkylating molecules that react with bionucleophiles like nucleic acids, thus can interfere with DNA synthesis but on the other hand they exhibit unselective protein binding [13, 14]. Since alkylating compounds have been a cornerstone for cancer therapy and the structures of illudin M and S differ from conventional chemotherapeutics, they have been considered very promising molecules for the development of novel semisynthetic anticancer drugs [15, 16]. Illudin M has been reported to be produced by submerged cultivation of several *Omphalotus* strains including *O. nidiformis, O. olivascens* var *indigo* [17], *O. illudens* [11]*, O. japonicus* [18]*, O. mexicanus* [19] and *O. subilludens* [20]. Illudin M is secreted to the culture broth from where it is commonly extracted with organic solvents followed by column chromatography and crystallization [21].

During the development of the illudins as potential antitumor agents, it turned out that the natural compounds require chemical modifications for improved selectivity and efficacy in order to bring them into clinical practice [16]. For the illudins M and S there are well established semisynthetic routes to generate acylfulvenes with fine-tuned pharmacological properties which allow a specific targeting of cancer cells [15]. In an attempt to produce improved illudin derivatives (hydroxymethyl)acylfulvene a semi-synthetic illudin S derivate also known as Irofulven was developed but had to be withdrawn in 2012 from clinical trials due to efficacy issues [22]. However it is currently undergoing phase II clinical trials being carried by Allarity^®^ therapeutics where it is been tested for the treatment of castration-resistant metastatic prostate cancer in an improved treatment regimen [23–26].

In other studies, derivatives based on illudin M have been developed by semi synthetic approaches and several ester conjugates showed improved specificity when comparing fibroblasts against malignant cells from pancreatic and colon adenocarcinoma [21], and, two ferrocene esters of illudin M displayed improved selectivity towards melanoma cells [27]. Furthermore, some metallocenedicarboxylates exhibited 40 times less toxicity and improved selectivity against several cancer cell lines including tumor lines derived from colon, pancreas, breast, melanoma, cervix, and leukemia; showing additionally stability under physiological conditions and tolerable toxicity profile when evaluated in rodent models by repeatedly administering high doses [28]. Such findings encourage follow up studies and these promising molecules could undergo further pre-clinical development. However, semi synthesis of these derivatives requires sustainable supplies of the natural compound in multi-gram scale [29], with purities ≥ 95% for further derivatization [28]. Although a supply of Illudin S has been established from natural sources [30], to the best of our knowledge published details regarding media composition and culture conditions are lacking and reports describing the scalable production of illudin M are not available. We therefore intended to develop a process for the production of illudin M in order to support medicinal chemistry studies and further preclinical development.

Process development commonly starts with small scale experiments in microtiter plates and shake flasks, which allow medium to high throughput experiments and assessment of important parameters that can be used for subsequent process transfer into stirred tank bioreactors and further scale-up [31]. Volumetric power input, mixing time and oxygen transfer rate (OTR) are common scale-up parameters for which empirical correlations have been determined in shake flasks [32–34] and especially the maximum local energy dissipation rate has been proposed as suitable criteria for scale-up of filamentous cultivations due to the strong influence on morphology [35, 36]. In the case of fungal cultivations, scale-up is not a straightforward process since morphology development in submerged cultures is difficult to predict, depends on many process parameters and has a great impact on productivity [35]. Even though, several approaches such as inoculum concentration, pH and pH shifting, mechanical stress (volumetric power input, micro and macro-particle addition) and osmolality have been used for controlling fungal morphology on the process level; intrinsic properties of the strain play a role in productivity and therefore assessment of cultivation parameters often relies on empirical trials [37–39].

This work describes a sequence of experiments that improved the titers of illudin M in shake flasks by focusing on identification of critical aspects influencing product formation. The stepwise improvement of illudin M titers was achieved by following a hypothesis-driven approach that allowed identification of a trend towards higher production of the target molecule with a moderate number of experiments. Conclusions drawn from previous experiments were consistently confirmed or rejected by results of carefully planned follow up experiments, leading to an improved shake flask process. This is the first paper of a series of reports dealing with the biotechnological production of illudin M [40]. The aim of the present work was the improvement of illudin M titers at shake flask level; and while hypotheses had been developed to design follow-up experiments it was not scope of this publication to prove the underlying physical and biochemical basis in detail, when such experiments where successful.

## Results

### Development of analytical methods

To enable rapid monitoring of illudin M in a high number of samples, a fast and reliable method to quantify titers was developed. Since the compound is secreted to the medium and does not adhere to biomass, the latter was removed by centrifugation. Therefore, a quick extraction protocol using a small volume of supernatant was established, followed by separation of the crude mixture using reverse-phase high-performance liquid chromatography (RP-HPLC) coupled with diode array detection (DAD). The extraction procedure and subsequent separation were optimized so that results were obtained within a few minutes after sampling, enabling medium-throughput analyses. To develop and verify this method, a small quantity of illudin M was purified and subsequently analyzed using high-resolution mass-spectrometry (HRMS) and nuclear magnetic resonance (NMR) spectroscopy to confirm the purity, structure and the retention time of the molecule [40].

### Media screening

**Fig. 1:**
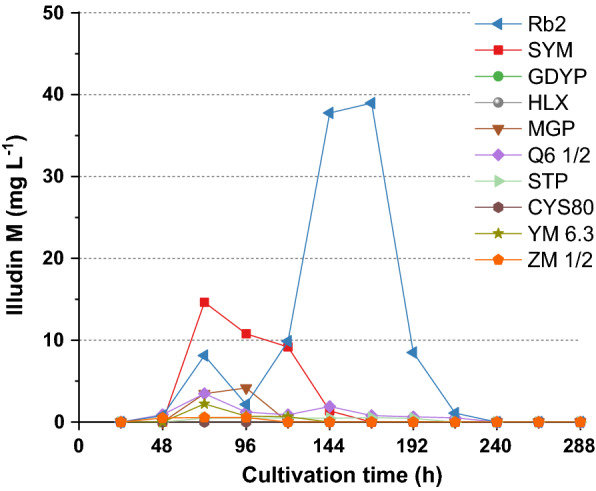
Illudin M kinetics from cultures of *O. nidiformis* cultivated in different media. Single cultures were prepared according to method SP1 using ten different complex media. Illudin M titers were derived from cell free culture supernatant

*Omphalotus nidiformis* was selected for initial screening of a panel of standard culture media consisting of different complex components. The strain was cultivated according to method SP1 and incubated for 336 h to evaluate substrate consumption and product formation. Production kinetics were obtained by sampling every 24 h (for explanation of SP1 to SP4, see “Methods” section: seed preparation). Figure 1 shows the kinetics of illudin M production in this experiment. The highest production (~ 40 mg L^−1^) under these conditions was achieved with the medium Rb2 (see Table 1), which contains corn steep solids (CSS). Since our results were consistent with reported findings, the medium Rb2 was selected for further experiments.

**Table 1:** Highest product titers during media screening

Culture medium	Illudin M (mg L^−1^)
Rb2	40
SYM	15
GDYP	Not detected
HLX	Not detected
MGP	4
Q6 ½	4
STP	1
CYS80	Not detected
YM 6.3	2
ZM ½	1

The titers of illudin M shown in the kinetics of all experiments in this work are the concentrations of product in cell free supernatant since biomass and insoluble medium components were removed by centrifugation prior the extraction of the samples. However, during optimized cultivations the amount of wet biomass was determined at harvest in order to calculate the volumetric productivity of the cultures. Biomass never exceeded 6% of the total volume of the culture.

### Screening of producer strains

Six other strains of the genus *Omphalotus,* which have been reported to produce illudins were obtained. All strains were prepared according to method SP1 and cultivated under identical conditions in Rb2 medium for 336 h. Kinetics for illudin M production were established by sampling every 24 h. The performance of the cultivations was monitored by analysis of product formation and substrate kinetics.

**Fig. 2:**
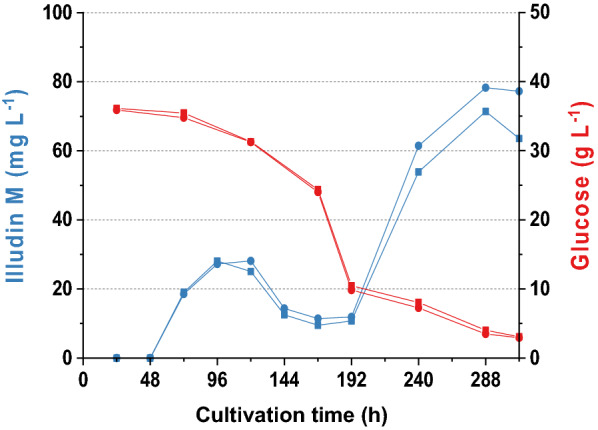
Product and substrate kinetics of *Omphalotus nidiformis* cultivated in Rb2 medium. Illudin M titers (blue) and glucose (red) concentration over the cultivation time. The experiment was carried out in duplicates. Illudin M titers were derived from cell free culture supernatant

All strains exhibited pelleted growth during the submerged cultivation in shake flasks. The highest titer of illudin M (78 mg L^−1^) was achieved with *O. nidiformis* after 288 h of cultivation(see Table 2). Interestingly, the analysis of the *O*. *nidiformis* kinetics indicated two distinct peaks of illudin M production (see Fig. 2: 96 h, 288 h), a phenomenon, which was not observed with any of the other strains. Product and substrate kinetics of all strains are illustrated in Additional file 1 Fig. S1. Based on highest product titers, *O. nidiformis*, was selected for further investigation.

**Table 2:** Highest product titers during strain screening

Producer strain	^a^Illudin M (mg L^−1^)
*Omphalotus nidiformis*	71
*Omphalotus olearius* CBS488.95	10
*Omphalotus olearius* CBS102283	24
*Omphalotus subilludens*	18
*Omphalotus japonicus*	10
*Omphalotus olivascens* var. *indigo*	16
*Omphalotus mexicanus*	18

^a^Titers are an average of the highest titers of the duplicates

**Fig. 3:**
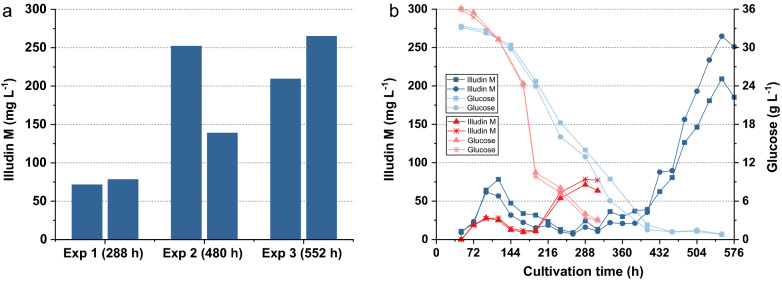
Variability of illudin M concentrations between different experiments with *O. nidiformis* cultivated in Rb2 medium according to method SP1. **a** Highest illudin M concentration of three independent experiments which were carried out in duplicates. Highest titers were achieved at different cultivation times (288 h; 480 h and 552 h). Experiment 2 showed a higher variance in illudin M titer than the other two experiments. **b** shows the kinetics for illudin M and glucose of Exp 1 (Red) and Exp 3 (blue). Illudin M titers were derived from cell free culture supernatant

Follow up experiments were made to investigate production at two distinct time points, finding that a similar production pattern occurred in all experiments but with variable time points and product titers as shown in Fig. 3.

We hypothesized that variability between these experiments (Fig. 3) could be related to the inoculum preparation since the liquid culture of this strain was a heterogeneous mixture due to the pelleted growth. The homogeneity of the inoculum prepared according SP1 depended on the efficiency of the homogenization step and influenced the pellet size in the liquid seed cultures. We observed that replicates from the same seed culture in some cases showed similar outcomes in product titers and consumption of substrates but higher variations were observed between separate experiments in which seed culture had been prepared independently. This becomes evident in Fig. 3b where the second peak of production is shifted more than 264 h in experiment 3. We concluded that seed preparation had a major impact on process performance and that this procedure must be standardized. Comparing the two kinetics in Fig. 3b, the consumption of glucose indicated that glucose depletion might play an additional role in product formation since the highest product titers occurred when the glucose concentration was low or depleted. Since activation of secondary metabolism upon depletion of a carbon source is a commonly observed phenomenon in fungal cultivations, we predicted that using a medium with reduced amount of glucose (G20/C5) would shift the second production peak to an earlier time point due to an earlier depletion of glucose and consequently overcoming a potential carbon catabolite repression.

### Development of seed culture

**Fig. 4:**
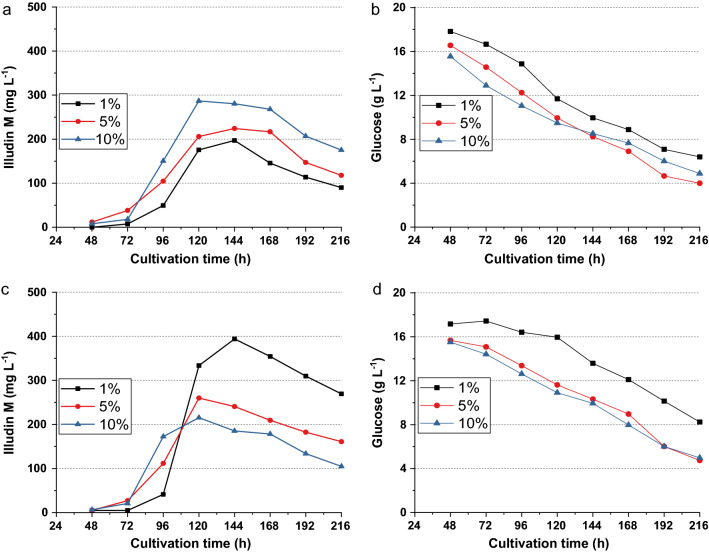
Illudin M and glucose kinetics from cultures of *O. nidiformis* prepared with two different inoculation procedures. All runs were performed as a single experiment. **a** Illudin M and **b** glucose concentrations of experiment 1 prepared according method SP1 (non-homogenized seed culture); **c** illudin M and **d** glucose concentrations of experiment 2 prepared according to method SP2 (homogenized seed culture). Both experiments were performed simultaneously. Inoculation was performed with an inoculation volume of: 1% (black), 5% (red) and 10% (blue). All cultures were prepared with medium G20/C5. Illudin M titers were derived from cell free culture supernatant

With the medium G20/C5, we simultaneously investigated the influence of three parameters on production: reduced glucose concentration, inoculum size and inoculum homogeneity. Therefore, three cultures were prepared according SP1 inoculating production cultures with 1%, 5% and 10% (V/V). Three other cultures were prepared according to SP2 and the production cultures were inoculated with 1%, 5% and 10% (V/V). Figure 4 illustrates the outcome of this experiment, which confirms that the reduction of glucose shifted production to an earlier stage. Although glucose was in the range of > 10 g L^−1^ as shown in Fig. 4b and d this reduction had a clear influence on titer and time point. It seems that both production phases fell together and product titers accumulated with the outcome that the highest experimental titer could be measured earlier than in previous experiments. As a consequence of the reduced glucose consumption in the culture with the highest titer, the initial glucose concentration was further reduced to 12 g L^−1^ in subsequent experiments.

Product kinetics with different amounts of inoculum indicated that optimal inoculum size for productivity might be a function of biomass and homogeneity. Figure 4a shows the overlay of three kinetics achieved with a non-homogenized seed culture, indicating an increase in product titers when the amount of inoculum was increased. Looking at Fig. 4c, where seed culture was homogenized prior inoculation, indicates the opposite: the lower the inoculum volume, the higher the final titer.

**Fig. 5:**
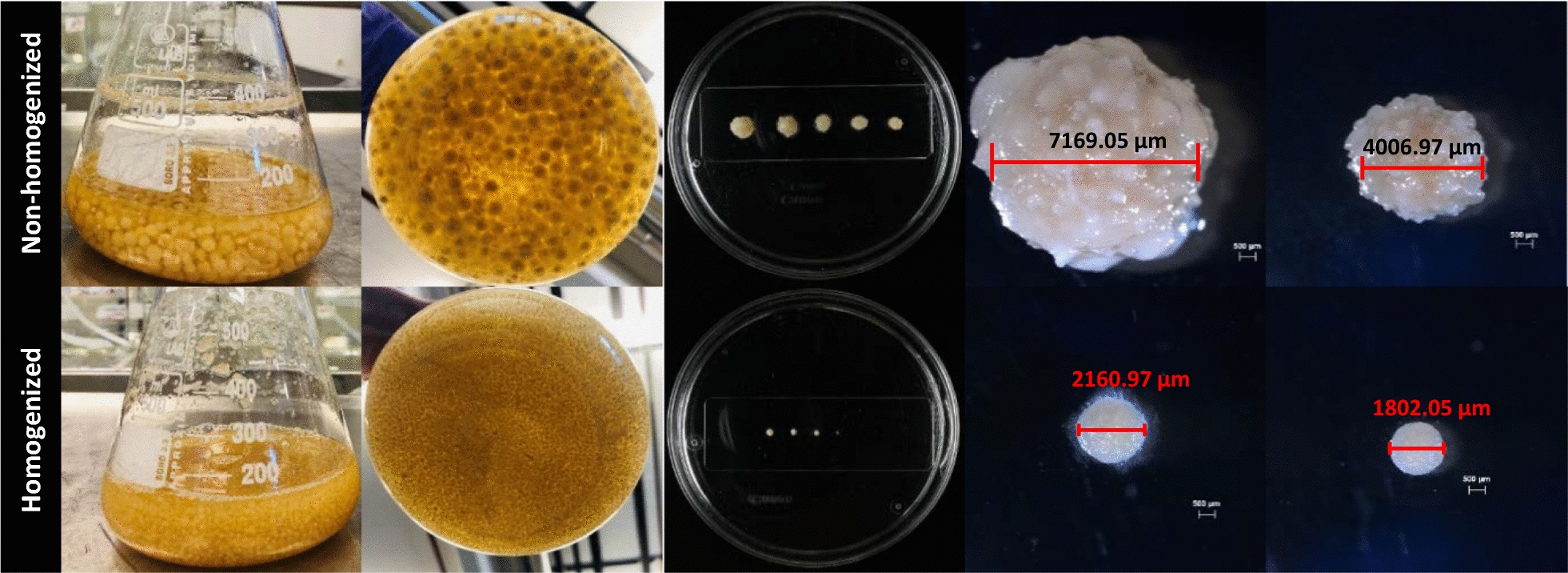
Pellet morphology after 144 h in cultures of *O. nidiformis* cultivated in G20/C5 medium. The upper row shows pellet appearance and size from main cultures prepared according to method SP1 inoculated at 10%. Lower row shows pellet appearance and size from main cultures prepared according method SP2 inoculated at 1%

In general, we observed that the homogenization resulted in a different appearance of the culture morphology. A comparison of two experiments is shown in Fig. 5 and it is evident that homogenization prior to inoculation led to a greater number of smaller pellets compared to the non-homogenized inoculation. This outcome highlights two positive aspects of homogenization of seed culture, on one hand smaller pellets in the main culture seemed to be better for production of illudin M and on the other hand, reduced inoculation volumes can ease the seed train in further process scale up.

Since with homogenized cultures larger inocula were detrimental for production of illudin M, we concluded that an optimal amount of active biomass must be obtained through a careful seed preparation, which results in an optimal number of active cells during the production phase.

**Fig. 6:**
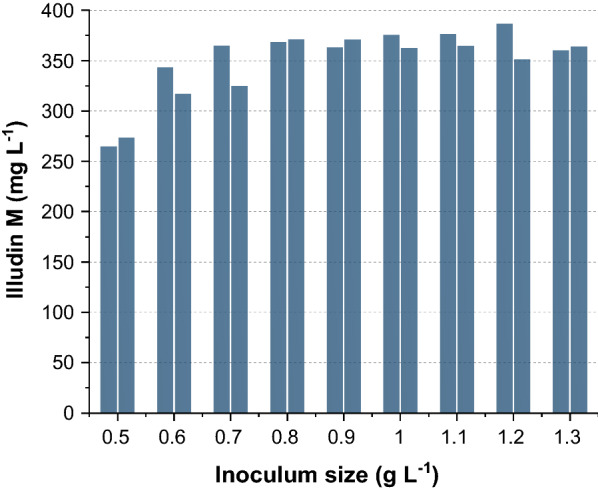
Influence of inoculum size on maximum product titers. The bars indicate the highest concentrations of illudin M measured after 144 h in duplicated cultures of *O.* *nidiformis* inoculated with different amounts of biomass. Cultures were prepared according to method SP3. Illudin M titers were derived from cell free culture supernatant

In order to avoid high variability between experiments it was necessary to establish a strictly standardized procedure. To standardize the procedure homogenized biomass was adjusted to a concentration of 200 g L^−1^ (Method SP3). From this defined seed preparation, nine duplicate experiments were performed to investigate the influence of different inoculum sizes on the illudin M titer (see Fig. 6).

In Fig. 6 the highest titers of each experiment are plotted, which were reached in all cultures at 144 h. The lowest concentrations with a final value of 0.5 g L^−1^ resulted in the lowest productivity and a higher final concentration of inoculum resulted in better production. Beyond 0.8–0.9 g L^−1^ of final biomass concentration no improvement in productivity could be observed and until 1.3 g L^−1^ no significant reduction in production due to higher biomass concentrations was observed.

**Fig. 7:**
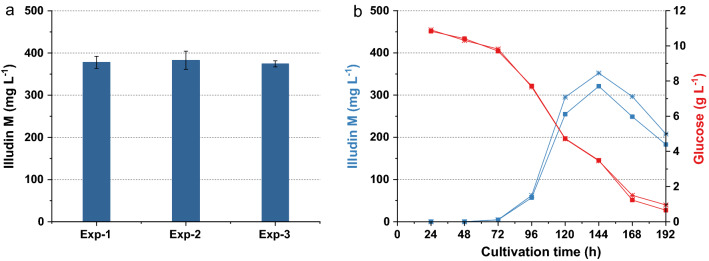
Variability of illudin M concentrations between different experiments with *O. nidiformis* cultivated in G12/C6 medium prepared according to method SP3. **a** Titers and variance of highest illudin M concentration of three independent experiments (five replicates each, error bars indicate ± 1 standard deviation from the mean). **b** shows the typical kinetics for product (blue) and substrate (red) of an experiment carried out in duplicates. Illudin M titers were derived from cell free culture supernatant

The concentration of the inoculum was set to 1 g L^−1^ and the seed culture was prepared accordingly. To measure the variance in production with this inoculation procedure several experiments in G12/C6 medium were conducted. We achieved a stable and reproducible titer of ~ 380 mg L^−1^ of illudin M after 144 h with low variances within 5 replicates in one experiment and between three separate experiments (Fig. 7a). The kinetics for product and substrate of typical cultivations with the medium G12/C6 are shown in Fig. 7b which are comparable to the kinetic presented in Fig. 4c when the culture was inoculated at 1% (v/v). The ANOVA of the data derived from these three independent experiments is shown in Table 3. The probability Pr(> F) higher than 0.05 indicates that there are no significant differences between the groups, which confirms the reproducibility of the method and the results can be used for combined statistical analysis (see Table 9).

**Table 3:** ANOVA of three independent experiments with medium G12/C6

	df	SS	MS	F value	Pr> F
Run	2	137.6	68.82	0.287	0.756
Residuals	12	2879.2	239.94		

*df* degree of freedom, *SS* sum square, *MS* mean square, *Pr> F* probability value

### Medium optimization

Having defined standard operating conditions including age of the plates for seed culture preparation, cultivation time of the seed culture and inoculum preparation, we designed an experiment to investigate the optimal composition of glucose and corn steep solids in the culture medium. The factors investigated and their levels are listed in Table 4.

**Table 4:** Factors influencing illudin M formation and their levels

Factor	Symbol	Level				
		− 1.0	− 0.5	0	0.5	1.0
Corn steep solids (g L^−1^)	x1	3	Na	6	Na	9.0
Glucose monohydrate (g L^−1^)	x2	6	9	12	15	18.0

*Na* not applicable

The experimental space was set around the concentrations of the already tested medium G12/C6 (glucose monohydrate 12 g L^−1^; CSS 6 g L^−1^) with eleven data points. We focused on the uniform distribution around this established point rather than on a classical experimental design to exclude sudden drops in productivity, which are difficult to model. Those were expected by reduced growth and production due to nitrogen and/or phosphate limitation at low corn steep solids concentrations or ammonia intoxication by deamination of amino acids at high concentrations. For high glucose concentrations we did not expect a significant drop in productivity since this had been tested in earlier media (see Fig. 4), for reduced glucose concentrations a drop in productivity based on reduced growth was expected. Since high experimental variability was not expected due to the standardized seed preparation (see Fig. 7), the number of experiments was reduced to the absolute minimum. Table 5 presents the experimental design and the tested concentrations of CSS and glucose and the measured and predicted values of illudin M. These data were used to build a second order model using the rsm package (version 2.10.3) together with R (version 4.1.1). The performance of the model is represented by Eq. 1:

**Table 5:** Experimental design matrix describing illudin M production in response to medium components

Trial	Factor 1 (CSS g L^−1^)		Factor 2 (glucose g L^−1^)		Response	
					illudin M (mg L^−1^)	
	Actual	Coded	Actual	Coded	Actual	Predicted
1	3	− 1	9	− 0.5	183	185
2	3	− 1	12	0	207	204
3	3	− 1	15	0.5	192	193
4	6	0	6	− 1	265	266
5	6	0	9	− 0.5	318	322
6	6	0	12	0	365	349
7	6	0	15	0.5	333	345
8	6	0	18	1	315	312
9	9	1	9	− 0.5	272	264
10	9	1	12	0	283	298
11	9	1	15	0.5	310	302

The cultures were prepared according to method SP4. The highest titer was measured at the central point of the experimental space G12/C6

1 $${c}_{illudin M}\,=\,348.80+46.98x1+23.10x2+15.28x1x2-97.86{x1}^{2}-59.59{x2}^{2}$$

With a stationary point at x1 = 0.258 and x2 = 0.227 (CSS = 6.77 g/L and glucose = 13.36). The multiple R^2^: 0.9777 and the adjusted R^2^: 0.9555 indicate a good fit of the model which is confirmed by the F-statistic with a p-value = 0.00039.

The measured data was analyzed by ANOVA using R (version 4.1.1). The data presented in Table 6 indicates as expected the significance of glucose monohydrate and CSS on illudin production Pr > F of first order (FO) and pure quadratic interactions (PQ) are lower than 0.05, whereas there seems to be no significant interactions of both factors (TWI > 0.05).

**Table 6:** Analysis of variance (ANOVA) for illudin M production in response to medium components

	df	SS	MS	F value	Pr > F
FO	2	15,110.1	7555.1	46.9401	0.0005750
TWI	1	233.3	233.3	1.4497	0.2824656
PQ	2	19,992.2	9996.1	62.1064	0.0002946
Lack of fit	5	804.8	161.0		

*df* degree of freedom, *SS* sum square, *MS* mean square, *Pr> F* probability value

**Fig. 8:**
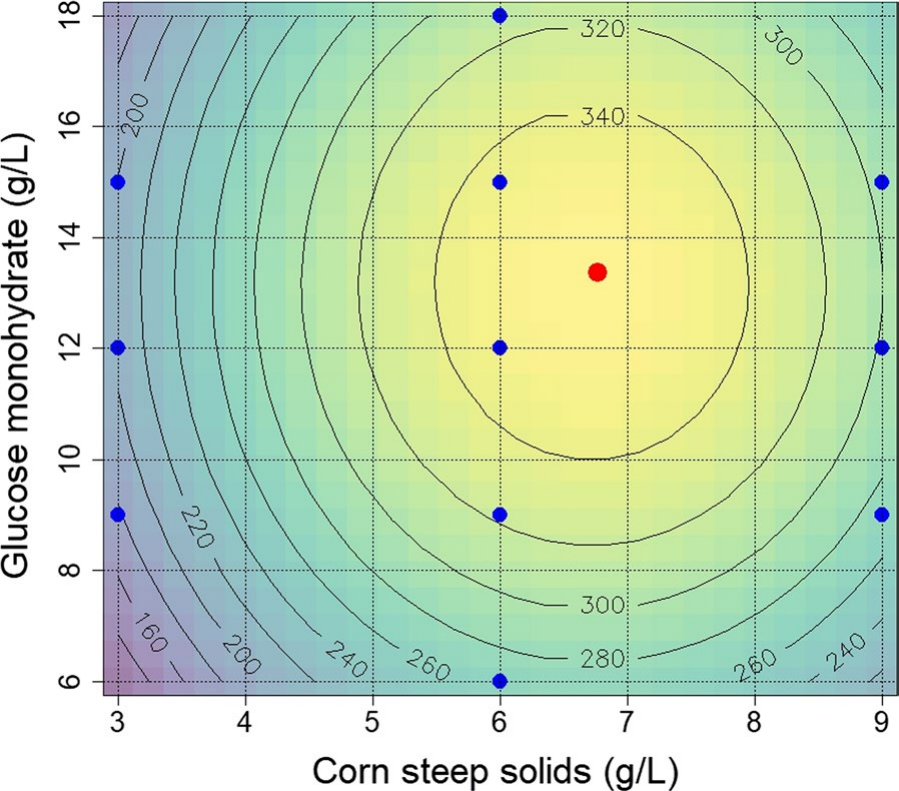
Contour plot derived from experimental data illustrating the influence of different concentrations of glucose and corn steep solids on illudin M titers at 144 h. Background colors from blue to yellow indicate increasing illudin M titers, underlined by grey contour lines derived from the second order model build in R with the rsm package. Model summary: Multiple R^2^: 0.9777; Adjusted R^2^: 0.955; F-statistic: 43.91 on 5 and 5 DF; p-value: 3.92e-04. The rsm package calculated a stationary point (maximum) at Glucose 13.36 g L^−1^ and corn steep solids at 6.77 g L^−1^ highlighted with the red dot. Blue dots indicate the nutrient composition of the experiments. Cultures were prepared with *O. nidiformis* according to method SP4

To illustrate the main effect of glucose monohydrate and CSS concentrations the second order model was plotted as 2D contour graph, representing the illudin M concentration (response) as a function of glucose and CSS concentration. The concentrations analyzed to establish the model are indicated in blue and the calculated maximum is indicated in red (Fig. 8).

**Fig. 9:**
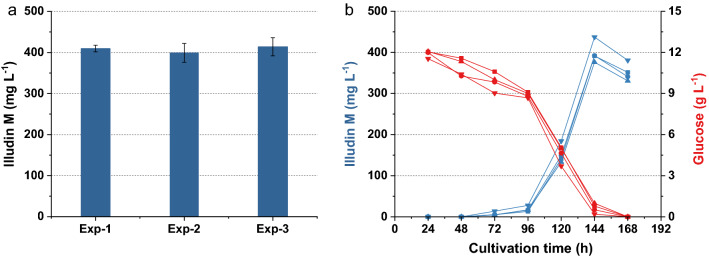
Variability of illudin M concentrations between different experiments with *O. nidiformis* cultivated in G13.5/C7 medium prepared according to method SP4. **a** Titers and variance of highest illudin M concentration of three independent experiments (four replicates each, error bars indicate ± 1 standard deviation from the mean). **b** shows the typical kinetics for product (blue) and substrate (red) of one of these experiments. Illudin M titers were derived from cell free culture supernatant

The final base medium for production was adjusted to 13.5 g L^−1^ of glucose monohydrate and 7.0 g L^−1^ corn steep solids. Interestingly, regardless of the concentration of glucose and corn steep solids, all cultures reached the highest product concentration at 144 h. This implies that the trigger or “window of product formation” was a simultaneous event in all cultures but the titers achievable were a function of the active biomass and the concentration of nutrients available in the production phase. To confirm the titers achievable with G13.5/C7 base medium and to verify reproducibility, several experiments were conducted at different time points with low variation observable (see Fig. 9). The ANOVA of the data derived from these three independent experiments is shown in Table 7. The probability Pr> F higher than 0.05 indicated that there were no significant differences between the groups and the results were used for combined statistical analysis (see Table 9).

**Table 7:** ANOVA of three independent experiments with medium G13.5/C7

	df	SS	MS	F value	Pr> F
Run	2	464	232.1	0.488	0.629
Residuals	9	4284	476.0		

*df* degree of freedom, *SS* sum square, *MS* mean square, *Pr> F* probability value

### Development of precursor feeding strategy

The depletion of glucose in cultures prepared according to SP4 seemed to correlate with a decline in measured product titers (Fig. 9b), which was in contrast to earlier experiments were inoculum was prepared differently (Fig. 7b). To investigate if this drop in product titer was due to lack of glucose, several feeding experiments with glucose were performed, which didn’t result in higher productivity (see Additional file 1: Fig. S2). Therefore, glucose was ruled out as a substrate for a prolonged production phase, yet seemed to be beneficial to maintain further growth and energy supply.

**Fig. 10:**
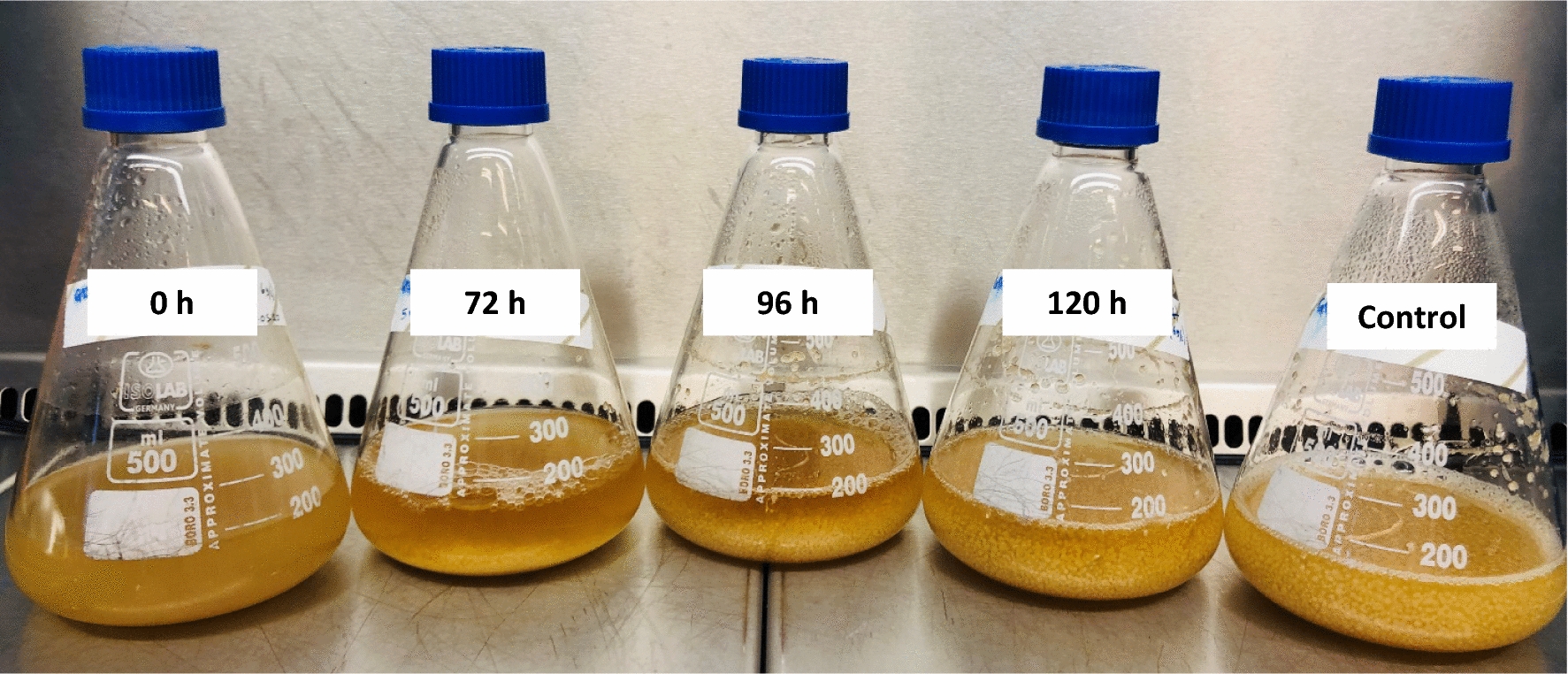
Cultures of *O. nidiformis* cultivated in G13.5/C7 medium after acetate feed at different time points. Cultures were fed at the indicated time to reach a final concentration of 8 g L^−1^ acetate (CH_3_CO_2_K). The picture taken at 168 h of cultivation time illustrates the influence of the feeding time on biomass growth

A second approach to overcome a potential biosynthetic bottleneck was the feed of acetate. To investigate this, acetate derived from CH_3_CO_2_K was fed to cultures prepared according SP4. The CH_3_CO_2_K solution was fed at 0; 72; 96 and 120 h to reach a final concentration of 0.13 M (equal to 8 g L^−1^ acetate) in the cultures. Cultures fed at 0 h did not grow, cultures fed at 72 h showed poor growth, in cultures fed at 96 h growth could be observed and at 120 h of addition, the growth was almost comparable to the control without acetate addition. The feed of acetate and the time of feeding had a direct impact on cell growth and the cell density achieved over the cultivation time (see Fig. 10).

In the cultures fed at 0 h and 72 h no product was detected and therefore the sampling was stopped after 168 h. The highest titers were measured in the cultures fed at 96 h (> 800 mg L^−1^) and slightly lower titers were measured after feeding at 120 h. The control without feeding produced similar illudin M titers as expected from previous experiments. The comparison of process kinetics shows that all cultures reached the highest titer between 168 and 192 h meaning that the window of product formation was prolonged in the new setup when compared with the control experiment (see Additional file 1: Fig. S3).

**Fig. 11:**
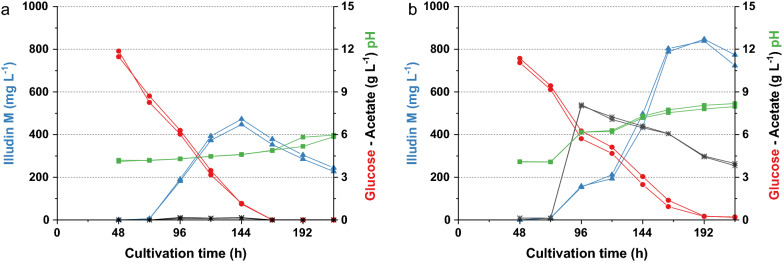
Comparison of cultivation kinetics of cultures of *O. nidiformis* with and without feed of acetate (CH_3_CO_2_K). **a **Substrates, pH and product kinetics of standard batch cultivations without feed **b** Substrates, pH and product kinetics of cultures fed with acetate to reach a concentration of 8 g L^−1^ at 96. The curves are colored according to the labels of each axis. All experiments were conducted in G13.5/C7 medium. Cultures were prepared according to method SP4. Illudin M titers were derived from cell free culture supernatant

The plotted pH in Fig. 11a and b indicated that the feed of the basic potassium-acetate solution had an immediate impact on culture pH, which increased from pH 4.2 to pH 6.2, reaching pH 8.0 at the end of the experiment, while the control reached pH 4.6 at that time. The increase in pH was critical since an identical shake flask experiment where the acetate feed was buffered to maintain pH 4.2, led to severe growth inhibition reflected in no consumption of substrates and no production of illudin M (see Additional file 1: Fig. S4).

Based on these findings we concluded that the impact of the feeding of potassium acetate on biomass development and production can be connected to the change in pH, the acetate or can be a combined effect of both. The observed impact on biomass growth (see Fig. 10) might also contribute to an “optimal” ratio of active biomass to nutrients when production starts. Another observation is that illudin M titers declined once glucose was depleted despite acetate being available in the culture broth. The results do not allow conclusions about the influence of the potassium ion on production.

**Fig. 12:**
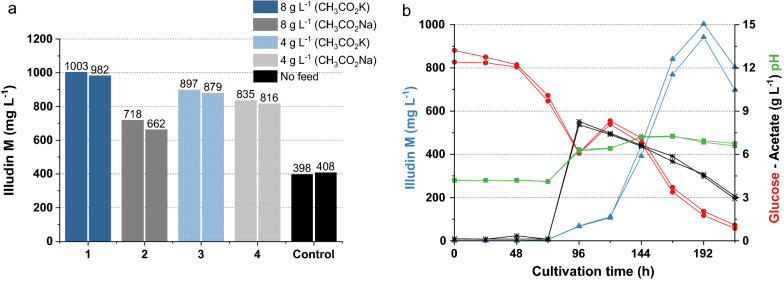
Product titers of cultures of *O. nidiformis* fed with two diferent acetate sources (CH_3_CO_2_K; CH_3_CO_2_Na) and kinectics of cultivation wit best illudin M production. **a** Barplot of the highest titers measured in the different cultures: bars in blue represent cultures fed with CH_3_CO_2_K: dark blue bars 8 g L^−1^ at 96 h and dotted light blue two times feed of 4 g L^−1^ (96 h and 168 h). Bars in gray represent cultures fed with CH_3_CO_2_Na: dark gray 8 g L^−1^ at 96 h and dotted ligh gray two times feed of 4 g L^−1^ (96 h and 168 h). All cultures were additionally fed with glucose (total addition 6 g L^−1^). **b** Substrates, illudin M and pH kinetics of the cultures with highest titer; CH_3_CO_2_K 8 g L^−1^ was fed at 96 h and glucose 6 g L^−1^ was fed at 120 h. The curves are colored according to the labels of each axis. All cultures were prepared using G13.5/C7 medium according to method SP4. Illudin M titers were derived from cell free culture supernatant

A new experiment was conducted using cultures prepared according to SP4. In this setup we investigated the influence on production of two different initial concentrations of acetate feed (4 g L^−1^ and 8 g L^−1^) coming from two different acetate salts (CH_3_CO_2_K and CH_3_CO_2_Na). The acetate feed was performed at 96 h and an additional glucose feed (total addition of 6 g L^−1^) at 120 h to rule out glucose depletion as cause of interruption of illudin M formation. The highest titers in all cultures were detected between 168 and 192 h (see Additional file 1: Fig. S5) and the best production was obtained in cultures fed with 8 g L^−1^ of CH_3_CO_2_K and a feed of 6 g L^−1^ of glucose as shown in Fig. 12a. The pH shifted in all cases from pH 4.2 to pH 6.2. The product titers increased in comparison with the previous experiment with highest titers reaching up to ~ 1000 mg L^−1^ of illudin M, indicating that glucose might serve as energy source for biosynthesis of the target molecule in this prolonged production phase. With this new setup, we observed that glucose was rapidly consumed despite the feed at 120 h and it was nearly depleted by the time that the illudin M titer reached its maximum (see Fig. 12b).

**Fig. 13:**
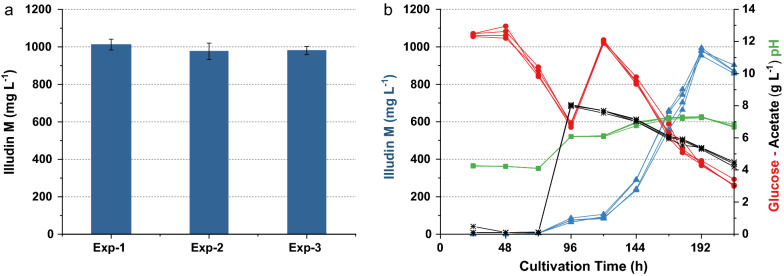
Variability of illudin M concentrations between different experiments with *O. nidiformis* cultivated in G13.5/C7 medium prepared according to method SP4. **a** Barplot of titers and variance of highest illudin M concentration at 192 h of three independent experiments (four replicates each, error bars indicate ± 1 standard deviation from the mean). A single feed of CH_3_CO_2_K (8 g L^−1^) was performed at 96 h combined with a single feed of glucose (total addition 6 g L^−1^) at 120 h, **b** shows the typical substrates, pH and product kinetics of one of these experiments. Curves are colored according to the color of the labels of each axis. To estimate the volumetric productivity, the titer measured in the cell free supernatant should be reduced by ~ 6% that is the maximal volume of biomass measured at harvest in these experiments

A final experiment was conducted to exclude low glucose concentration at the end of the production phase as the cause of decrease in production of illudin M. We evaluated if a two point feed of glucose would improve current titers. However, the excess of glucose and acetate at 192 h did not further prolong the production phase and the product titers declined after that time (see Additional file 1: Fig. S6). These results suggested that additional feeding would not increase the achievable titers and either the trigger of production was not present anymore or a self-inhibition or self-intoxication with the product occurred, which prevents the production of higher titers in the culture. Based on these results, the final cultivation and feeding strategy was established using a culture prepared according SP4. This culture was treated with a single feed of CH_3_CO_2_K (8 g L^−1^) at 96 h combined with a single feed of glucose (6 g L^−1^) at 120 h. This final process delivered stable illudin titers (> 950 mg L^−1^) in several independent experiments as shown in Fig. 13. The ANOVA of the data derived from these three independent experiments is shown in Table 8. The probability Pr> F higher than 0.05 indicated that there were no significant differences between the groups, the results were used for combined statistical analysis (see Table 9).

**Table 8:** ANOVA of three independent experiments with medium G13.5/C7 plus feed

	df	Sum Sq	Mean Sq	F value	Pr> F
Run	2	3042	1521	1.068	0.383
Residuals	9	12,812	1424		

*df* degree of freedom, *SS* sum square, *MS* mean square, *Pr> F* probability value

## Discussion

Illudin M is a potent anticancer lead molecule that has been produced from *Omphalotus* strains cultivated in several complex media. The highest titers were reported from cultures containing corn steep liquor or solids in the base media. During the discovery and evaluation of illudins, it was evident that the antimicrobial activity of culture extracts increased when the producer strain was cultivated in a medium containing corn steep liquor [11]; it was therefore concluded that this substrate could potentially enhance the production of the cytotoxic and antibacterial molecules. A comparison of studies reporting the production of the molecules indicated that the better results were obtained in media containing corn steep liquor or solids when compared with productivities in other media [15, 16, 18]. Corn steep liquor has been extensively used in microbiology as a main nitrogen source and seemed to be essential for the production of some fungal metabolites like penicillin and also the fungal cytotoxin pleurotin, yet the actual trigger for product formation was never identified despite extensive efforts [41–43]. We adapted a reported medium containing corn steep solids [10] and used it as starting point for optimization experiments to increase illudin M titers in submerged cultures of *Omphalotus nidiformis*. Concentrations of nitrogen and phosphate sources were not investigated analytically, since we assumed that optimal concentrations for growth and product formation would be determined by varying the composition of the two main medium components (glucose and corn steep solids). By reducing the initial high concentration of glucose in the culture medium, the peak of illudin M production occurred at an earlier time point. This phenomenon could be explained by the occurrence of carbon catabolite repression (CCR) since glucose is a rapidly metabolized sugar and often preferred over other carbon sources used for respiration and growth [44, 45]. This causes cells to produce specific enzymes to catabolize the fast metabolized sugars while repressing expression of other enzymes required for utilization of secondary substrates. Expression of the latter enzymes is upregulated when the preferred carbon source is depleted [46].

One of the critical points for characterization of the initial process was the high variability in production onset and product titers. Since these variations made interpretation of our findings difficult, we focused on the identification of key parameters causing these fluctuations. We identified that inoculum homogeneity had a major impact on reproducibility since the pelleted growth of the strain in the liquid medium led to heterogeneous seed cultures. We found that cultivations where big pellets formed, were detrimental for illudin M production, which was expected since it is been extensively discussed in literature that pellet morphology during submerged cultivation brings additional complexity—in contrast to cell suspensions—as the size of the pellets can influence the cultivation performance and productivity [35, 47, 48]. It is commonly accepted that the pelleted growth can cause nutrient gradients, i.e. oxygen limitation and nutrients depletion towards the center of the pellet, since the outer cells are depleting those nutrients. Some studies have stated that pellets exceeding a critical diameter might consist of active and growing cells restricted to the surface, and inactive or dead cells in the center; therefore, increasing amount of biomass in the course of a cultivation were big pellets are formed does not necessarily correlate with more active cells in the culture, but the opposite [49, 50].

It was therefore plausible that in our experiments the non-homogenized cultures with bigger pellets had reduced numbers of active cells compared with the homogenized cultures and that the number of active cells could be only moderately increased by a higher inoculum volume. In the case of a homogenized seed culture, a much higher amount of active cells was obtained since the disruption of the pellets into smaller fragments provided more starting points for cell growth, and the smaller pellet size contributed to better oxygen diffusion into the cells. In this sense, biomass quantification as parameter to evaluate growth kinetics, might be insufficient for explaining the growth phases of the cells, thus it would be more appropriate to look at the respiration activity which can be monitored in shake flasks with devices such as RAMOS (Respiration Activity MOnitoring System [51, 52].

**Fig. 14:**
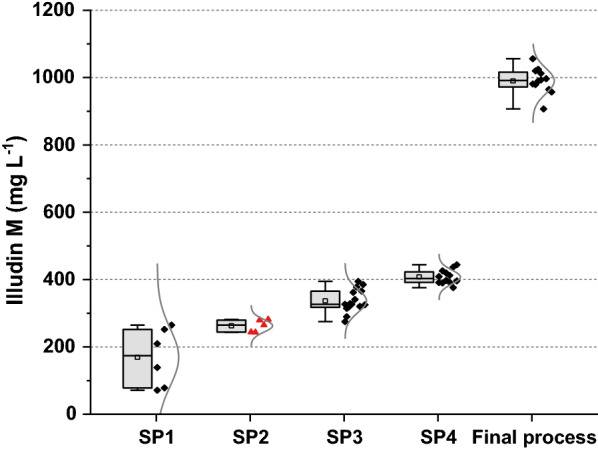
Box plots illustrating illudin M titers and experimental variation achieved with standard experiments conducted at different stages of this development campaign. SP1, SP2, SP3 and SP4 are four different methods for seed preparation. The final process was prepared using method SP4. SP2 (marked in red) was adapted after one experiment and further developed to SP3 and therefore independent experiments were not conducted and the variance illustrated is not directly comparable to the other datasets shown. The reduced variance at later stages is a result of the improved seed preparation. Higher titers achieved in later experimental stages are the results of changes in media composition (SP1 = Rb2, SP2 and SP3 = G12/C6, SP4 = G13.5/C7 and Final process = G13.5/C7 plus feed)

During cultivation of filamentous organisms, high variability on productivity can occur when the inoculum is prepared with vegetative biomass (e.g., mycelial mats) instead of spores [53]. Spores might be desirable for inoculum preparation due to better quantification and therefore better reproducibility but not all fungi can sporulate under laboratory conditions and spore preparation may be a slow process in many cases. As an alternative to inocula based on spores, some strategies like pellet-dispersion i.e. homogenization of seed culture, showed to be efficient for improved production of citric acid by *Aspergillus niger* with low variations in final product titers [54]. We followed a similar strategy and improved the reproducibility of our experiments by developing a careful inoculum preparation that consisted of dosing a defined amount of homogenized biomass into the cultures. A strict homogeneous seed culture becomes probably less important at larger scales since differences in culture homogeneity will average out with larger inoculation volumes. These improvements in reproducibility are illustrated in Fig. 14 were the influence of the different seed preparations on the variability of the target parameter is illustrated. By reducing the initially high variability of final titer and time of production we were able to quantify (with a low number of experiments) the influence of small experimental variations i.e. change in media composition from G12/C6 to G13.5/C7. This had been impossible at the beginning of the process development and we emphasize that only after strict standardization of the seed preparation, it was possible to have a meaningful measurement of the influence of the tested parameters on our dependent variable.

Table 9 lists the respective statistical values calculated for the experiments illustrated in Fig. 14. From the coefficient of variation, it is evident that the standardization of the seed preparation (SP1 to SP4) reduced the variability by a factor of 10. The investigation of method SP2 (indicated with^a^) was a single experiment in the transition from SP1 to SP3 thus the statistics in Table 9 do not represent independent experiments as for the others.

**Table 9:** Combined statistical analysis of different stages during medium development

	N total	Mean	SD	CV
SP1	6	169	85	0.50
SP2^a^	5	263	18	0.07
SP3	15	337	34	0.10
SP4	12	408	21	0.05
Final process	12	990	38	0.04

*N* number of samples, *SD* standard deviation, *CV* coefficient of variation

After achieving a stable batch process with improved titers, we observed a correlation between substrate depletion and a drop in production and concluded that a lack of biosynthetic precursors could be causal for that effect. Considering that illudin M is a sesquiterpene, fully derived from acetyl-CoA extender units, we developed a feeding strategy using acetate and glucose that more than doubled illudin M titers achieved with the previous batch process. Through the mevalonate pathway (MVA), acety-CoaA units are converted into isopentenyl diphosphate (IPP) and dimethyl-allyl diphosphate (DMAPP) that are precursors of terpenes biosynthesis in fungi [55]. By providing acetate at pH 6.2 to cultures of *O. nidiformis*, small quantities of undissociated acetate can penetrate the cell and reach the cytosol from where it is channeled as acetyl-CoA into the MVA pathway. Isopentyl pyrophosphate, the direct precursor of fungal terpenes, originates from this pathway [55, 56]. We assume that through direct provision of acetate (in form of acetyl-CoA) to the MVA pathway, we circumvented a biosynthetic bottleneck for isoprenoid biosynthesis in our process, since we avoided the energetically unfavorable channeling of glucose through the mitochondrion involving several transformation steps before conversion into cytoplasmic acetyl-CoA (see Additional file 1: Fig. S6).

It is plausible, that with glucose as the main carbon source, *O. nidiformis* had to divide resources between respiration, biomass build-up and secondary metabolism. Thus, when the secondary metabolism was starting to be upregulated the feed of acetate at 96 h and the additional feeding of glucose at 120 h were able to enhance the production of illudin M since *O.* *nidiformis* could utilize glucose for continued respiration and acetate for the biosynthesis of secondary metabolites.

Acetate has been considered as an economical substrate for biomanufacturing of diverse products which utilize acetyl-CoA as a biosynthetic precursor [57, 58]. Several studies focused on improved acetate utilization by increasing the tolerance of the producer strain by metabolic engineering since acetate is toxic for most organisms due to the pH dependent dissociation of acetate in the cytosol and changes of the intracellular pH, which affects important enzymatic reactions [59]. In our final process, the feed of potassium acetate increased the pH of the cultures reducing the concentration of undissociated acetate to a level tolerated by *O. nidiformis*; thereby avoiding the growth inhibition observed when the feed was buffered to maintain the typical low pH of the cultures. This indicates the potential of a pH-regulated acetate feeding to overcome the sensitivity of the producer, avoiding time consuming mutagenesis experiments*.* After adapting this feeding strategy, we reached a stable titer of ~ 1000 mg L^−1^ (~ 940 mg L^−1^ when corrected by ~ 6% of removed biomass) within 192 h. This equals a volumetric productivity (VP) of ~ 117 mg L^−1^ d^−1^ in shake flasks.

Our experiments indicated that not only the initial quantity of biomass at inoculation time was important but also the control of cell growth by addition of CH_3_CO_2_K at 96 h seems to be beneficial for the overall product titer achievable (see Fig. 10). Although the mechanism is not yet fully understood, there is strong evidence that the raise in pH in combination with the physicochemical properties of the acetate reduced the cell growth. This resulted in an improved biomass to nutrient and precursor ratio when the secondary metabolism was upregulated, leading to an optimized production profile between 72 and 192 h.

A general consideration at the beginning of the experiments was the transfer of findings from a shaken bioreactor into stirred tanks in follow up studies. Several parameters have been suggested in literature as guidelines for the scale up of fungal cultivations and empirical models have been established to calculate those in a wide range of cultivation conditions in shake flasks [32–34]. However, the slow growth of *O. nidiformis* and the long and variable cultivation times (> 550 h) during initial experiments, made large culture volumes necessary to avoid culture depletion by daily sampling. Despite the fact that large culture volume made application of parameter estimation models inapplicable, the possibility of online monitoring of respiration in shake flasks can provide necessary insights for scale-up purposes.

## Conclusions

Using published data and following a hypothesis-driven approach, we developed an improved shake flask process with the natural producer *O. nidiformis* resulting in stable titers of ~ 1 g L^−1^ of illudin M after 192 h. This is the initial step to develop a scalable biotechnological process in stirred tank bioreactors. We believe that our approach might be useful and transferable to other production processes with similar compounds from filamentous fungi. Our process has overpassed the highest reported titers of illudin M that stated ~ 250 mg L^−1^ after one-month cultivation of *O. japonicus* in a standing culture [18]. During our study, we identified key cultivation parameters such as inoculum preparation and the control of biomass growth during the process, which seems critical to achieve an ideal biomass/nutrients ratio in the production phase. Furthermore, the composition of the base medium was adapted to trigger the secondary metabolism of our producer, and once the biosynthetic machinery for illudin M assembly starts, an additional feed of biosynthetic precursor together with glucose as an energy source, increased productivity by a factor of two. We have achieved a final titer that is 25 times higher than that at the beginning of the optimization process. These findings allow a stable production of high titers of illudin M in shake flasks and are the base for further adaptation of those protocols to submerged cultivations in small-scale stirred tank bioreactors and subsequent scale-up studies.

## Methods

### Media and supplements

Culture media used in this work are listed in Table 10. The Rb2 medium is a modified version of the original medium formulated by William J. Robbins and colleagues [10]. Liquid media were prepared by weighing the required amounts of components directly into the flask and filling up to final weight of 200 g with deionized water minus the expected inoculum volume in grams. When greater batches of flasks with the same media were required, the medium was prepared in one batch and special care was taken to distribute media with insoluble components equally into those flasks by continuously stirring the media while distributing. For preparation of solid media 20 g L^−1^ of agar were added to the respective medium prior sterilization.

**Table 10:** Standard culture media and supplements

Culture medium	Components and concentrations (g L^−1^)
YM 6.3	Malt extract (10), Glucose (4), Yeast extract (4), pH 6.3
Q6 ½	Glucose (2.5), Glycerol (10), Cotton seed flour (5), pH 7.2
Rb2	Glucose monohydrate (40), Corn Steep Solids (5), NaNO_3_ (3), KH_2_PO_4_ (1), KCl (0.5), MgSO_4_.7H_2_O (0.5), FeSO_4_.7H_2_O (0.01), sucrose (40)
GDYP	Dextrin (40), Glucose (10), Yeast extract (4), Soy peptone (2), KH_2_PO_4_ (2), FeCl_3_.6H_2_O (2), MgSO_4_ .7H_2_O (0.5)
SYM	Sucrose (10), Yeast extract (5), Malt Extract (30), pH 6.3
ZM ½	Molasses (5), Oat flour (5), Sucrose (4), Mannitol (4), Glucose (1.5), CaCO_3_ (1.5), Lactalbumin hydrolysate (0.5), (NH_4_)_2_SO_4_ (0.5), pH 7.2
MGP	Glucose (10), Maltose (20), Soy peptone (2), Yeast extract (1), KH_2_PO_4_ (1) MgSO_4_.7H_2_O (0.5), FeCl_3_ 10 mM (1 mL), ZnSO_4_ 11 mM (1 mL), CaCl_2_ 0.1 M (1 mL)
HLX	Sucrose (30), Casamino acids (10), K_2_HPO_4_ (1), Yeast extract (1), MgSO_4_.7H_2_O (0.5), KCL (0.5), FeSO_4_.7H_2_O (0.01)
CYS 80	Sucrose (80), Corn flour (50), Yeast extract (1)
STP	Sucrose (7), Tomato paste (10), Malt extract (5), (NH_4_)_2_SO_4_ (1), Soy flour (1), KH_2_PO_4_ (9)
G20/C5	Glucose monohydrate (20), Corn Steep Solids (5), Czapek-Dox Broth modified (35 mL)
G12/C6	Glucose monohydrate (12), Corn Steep Solids (6), Czapek-Dox Broth modified (35 mL)
G13.5/C7	Glucose monohydrate (13.5), Corn Steep Solids (7), Czapek-Dox Broth modified (35 mL)
Czapek-Dox broth modified	NaNO_3_ (1), KH_2_PO_4_ (1), MgSO_4_.7H_2_O (0.5), KCl (0.5), FeSO_4_.7H_2_O (0.01)

To prepare feeding stocks (Table 11) the required amounts of component was dissolved using approximately half of the final volume of water and mixed with a magnetic stirrer. In the case of glucose it was beneficial to heat up the water prior dissolving. The solution was completely transferred to a volumetric flask and filled up to the exact volume.

**Table 11:** Feed solutions

Feeding stock (g L^−1^)	Components and concentrations (g L^−1^)
Glucose (360)	Glucose monohydrate (396)
Acetate (600)	Potassium acetate (980)

### Strains

The producer strains are listed in Table 12 and were obtained from the German Collection of Microorganisms and Cell Cultures (DSMZ, Braunschweig, Germany) and from the Westerdijk Fungal Biodiversity Institute (WFBI, formely CBS, Utrecht, Netherlands).

**Table 12:** List of screened strains

Code	Taxon name	Origin
DSM23613	*Omphalotus nidiformis*	Australia
CBS102283	*Omphalotus olearius*	Netherlands
CBS 488.95	*Omphalotus olearius*	Austria
CBS660.85	*Omphalotus subilludens*	USA
CBS446.69	*Omphalotus japonicus*	Japan
CBS101447	*Omphalotus olivascens* var. *indigo*	Mexico
CBS101446	*Omphalotus mexicanus*	Guatemala

### Standard cultivation conditions

All procedures involving open cultures were done under laminar flow to prevent contamination.

Strains were maintained on YM 6.3 plates at 23 °C in darkness. New plates were prepared by placing an agar plug from a three weeks old plate in the middle of a fresh plate. All shake flasks experiments were conducted in 500 mL Erlenmeyer flasks sealed with membrane screw caps (Schott, Germany). The caps had an air inlet of 5 mm diameter, closed with an ePTFE membrane of 0.2 µm pore size. Liquid seed cultures were inoculated with agar plugs (7 mm diameter) generated with a sterile laboratory hole puncher. Cultures were homogenized with a Silent Crusher M (Heidolph, Germany). The liquid cultures (200 mL) were incubated at 23 °C and 160 min^−1^ shaking speed (orbit diameter 50 mm) in a Multitron shaker (Infors HT, Switzerland). Feeding was done by adding the required volume of feedstock. The required amount was calculated using Eq. 2:

2 $${V}_{FS}=\frac{{c}_{R}.{V}_{C}}{{c}_{FS}}$$

(where V_FS_ = Required volume of feedstock, c_R_ = required concentration of feed in the culture, V_C_ = culture volume, c_FS_ the concentration in the feedstock). The change in volume due to addition of stock solution was considered negligible since feedstocks were prepared as concentrated as possible to use minimal volumes.

### Seed preparation

*Method SP1* from two weeks old plates containing the producer strain, five agar plugs from the outer edge of the mycelium were added to liquid medium and homogenized at 5000 min^−1^ for 10 s. Cultures were incubated at standard conditions for seven days. This culture was subsequently used to inoculate main cultures (inoculation volume was 5% if not stated differently). Inoculum was added to the cultures using a sterile serological pipette.

*Method SP2* same procedure as method SP1 but at the end of the incubation time, the seed culture was homogenized a second time at 8000 min^−1^ for one minute. This culture was subsequently used to inoculate main cultures (inoculation volume was 1%, 5% and 10%).

*Method SP3* from two weeks old plates containing the producer strain, five agar plugs from the outer edge of the mycelium were added to liquid medium and homogenized at 8000 min^−1^ for one minute. The cultures were incubated for seven days. Afterwards the seed culture was homogenized at 8000 min^−1^ for one minute. For inoculum preparation, the homogenized seed culture was transferred into weighed sterile 50 mL Falcon™ tubes. Tubes were centrifuged at 4300×*g* for 15 min using an Eppendorf^®^ Centrifuge 5804R. After centrifugation, a pellet and a biomass containing top layer were observed. The liquid phase was carefully discarded to keep the complete biomass. The closed tubes were weighed again to determine the amount of biomass. Then, fresh culture medium was added to achieve a final concentration of 200 g L^−1^ biomass. The tubes containing the required volume of medium were homogenized for 10 s at 8000 min^−1^. The homogenized suspension was stirred at 500 min^−1^ with a magnetic stirrer during inoculation to ensure a homogenous distribution of biomass in the inoculum. Main cultures were inoculated at 1 g L^−1^ biomass unless stated differently.

*Method SP4* from three weeks old plates containing *O. nidiformis*, ten agar plugs from the outer edge of the mycelium were used for liquid seed cultures homogenizing at 8000 min^−1^ for one minute. Cultures were incubated for five days under standard conditions (23 °C, 160 min^−1^). Afterwards, the seed culture was homogenized at 8000 min^−1^ for one minute. The inoculum preparation was the same as in method SP3. Production cultures were inoculated at 1 g L^−1^ biomass and incubated under standard conditions.

### Final small-scale fed-batch process for illudin M production with *O. nidiformis*

For production of illudin M in shake flasks, seed and main cultures were prepared with 200 mL of G13.5/C7 medium in 500 mL Erlenmeyer flasks with membrane caps. The seed culture was prepared using method SP4. Main cultures were fed with potassium acetate (CH_3_CO_2_K) at 96 h to reach a concentration of 8 g L^−1^ and glucose was fed at 120 h to reach a final concentration of 6 g L^−1^. Cultures were harvested at about 192 h for further processing.

### Analytical methods

#### Product quantification

One sample of 1 mL of culture broth was centrifuged for 10 min at 16,800×*g*. From the clear supernatant 200 µL were mixed 1:1 with iced-cold acetonitrile and centrifuged for five minutes at 16,800×*g*. Subsequently, 100 µL were used for RP-HPLC analysis and the remaining was stored at − 20 °C. All measurements were performed with a Dionex UltiMate™ 3000 UHPLC System (Thermo Fischer Scientific™) equipped with a DAD detector on an Acquity UPLC® BEH C18 colum (1.7 μm, 2.1 mm × 50 mm, Waters™), at 40 °C. The mobile phase consisted of (A) H_2_O + 0.1% formic acid (FA) and (B) acetonitrile + 0.1% FA at a flow rate of 600 μL min^−1^. Separation of the 4 μL sample was achieved by a linear gradient initiated by a 0.5 min isocratic step at 5% (B) followed by an increase of (B) to 40% until 7.4 min. Subsequently the column was cleaned for the next injection by a linear increase to 100% (B) until 8.5 min, and an isocratic step at 100% (B) until 10.5 followed by a linear decrease to 5% (B) until 11.0 min, and equilibration with an isocratic step at 5% (B) until 13.0 min. The UV signal for quantification was set to 325 ± 10 nm. The calibration curve was established using a sample of the pure compound (> 95% purity). Pure compound was obtained and characterized using methods described in [40].

#### Substrates quantification

The sample was prepared with 200 µL of clear supernatant diluted 1:5 with and filtered through a Strata™-X 33 µm filter (Phenomenex, USA). The concentrations of acetate and glucose were monitored using an Agilent 1260 series HPLC and separating 2 µL of sample with an isocratic elution (2.5 mM sulfuric acid, 0.5 mL min^−1^) over a Phenomenex^®^ Rezex ROA-Organic Acid H + (8%) ion exchange column (300 mm × 7.8 mm × 8 μm) at 65 °C with a UV and RID detection. Calibration curves were prepared using pure standards.

#### High-resolution mass spectrometry (HR-MS)

High resolution electrospray ionization mass spectrometry (HRESIMS) was performed with an Agilent 1200 series HPLC–UV system combined with an ESI-TOF–MS (maXis^®^, Bruker) applying the analytical conditions described in the section for product analysis. Illudin M showed a molecular ion in the positive electrospray mode at m/z 271.1305 [M + Na]^+^ (calculated for C_15_H_20_NaO_3_^+^, 271.1307).

## Supplementary Information

**Additional file 1: Fig. S1.** Product and substrates kinetics of different *Omphalotus strains.* **Fig. S2.** Product and substrate kinetics from cultures of *O. nidiformis* fed with glucose at two different time points. **Fig. S3.** Illudin M, pH and substrate kinetics of cultures of *O. nidiformis* fed with acetate (CH_3_CO_2_K) at different time points. **Fig. S4.** Illudin M, pH and substrate kinetics from cultures of *O. nidiformis* fed with acetic acid. **Fig. S5.** Illudin M, pH and substrates kinectics from cultures of *O. nidiformis* fed with acetate from two different salts (CH_3_CO_2_K; CH_3_CO_2_Na). **Fig. S6.** Illudin M, pH and substrate kinetics from cultures of *O. nidiformis* fed with acetate and glucose. **Fig. S7.** General overview of glycolysis and the mevalonate (MVA) pathway in eukaryotes.

## Data Availability

All datasets generated during this study can be available from the corresponding author upon reasonable request.

## Abbreviations

RP-HPLC: Reverse phase high performance liquid chromatography
DAD: Diode array detector
HRMS: High-resolution mass spectrometry
NMR: Nuclear magnetic resonance
RSM: Response surface methodology
IPP: Isopentenyl-diphosphate
DMAPP: Dimethyl-allyl diphosphate
OTR: Oxygen transfer rate
