# Supplementary material for: Optimization of the production process for the anticancer lead compound illudin M: improving titers in shake-flasks
Source: Microb Cell Fact. 2022 May 28;21:98. doi: 10.1186/s12934-022-01827-z (PMC9148526; doi:10.1186/s12934-022-01827-z)
Supplement: Supplementary file 1 — Additional file 1: Fig. S1. Product and substrates kinetics of different Omphalotus strains. Fig. S2. Product and substrate kinetics from cultures of O. nidiformis fed with glucose at two different time points. Fig. S3. Illudin M, pH and substrate kinetics of cultures of O. nidiformis fed with acetate (CH3CO2K) at different time points. Fig. S4. Illudin M, pH and substrate kinetics from cultures of O. nidiformis fed with acetic acid. Fig. S5. Illudin M, pH and substrates kinectics from cultures of O. nidiformis fed with acetate from two different salts (CH3CO2K; CH3CO2Na). Fig. S6. Illudin M, pH and substrate kinetics from cultures of O. nidiformis fed with acetate and glucose. Fig. S7. General overview of glycolysis and the mevalonate (MVA) pathway in eukaryotes. [file 12934_2022_1827_MOESM1_ESM.docx]

# Additional file 1

Optimization of the production process for the anticancer lead compound illudin M I. Improving productivity in shake flasks

Lillibeth Chaverra-Muñoz^1, 2^, Theresa Briem^1, 2^, Stephan Hüttel*^1, 2^

1. Department of Microbial Drugs, Helmholtz Centre for Infection Research, Brunswick, Germany

2. German Centre for Infection Research (DZIF), Partner Site Hannover-Braunschweig, Brunswick, Germany.

Fig. S 1 Product and substrate kinetics of different *Omphalotus* strains cultivated in Rb2 medium. Illudin M titers (blue) and glucose concentration (red). Kinetics were carried out in duplicates using the strain: a *O. nidiformis*, b *O. mexicanus*, c *O. olearius* CBS102283, d *O. japonicus*, e *O. olearius*CBS488.95, f *O. subilludens* and g *O. olivascens*var *indigo*. Cultures were prepared according to the method SP1. Illudin M titers were derived from cell free culture supernatant.

Fig. S 2 Product and substrate kinetics from cultures of *O. nidiformis* fed with glucose at two different time points. Illudin M titers (blue) and glucose concentration (red). Glucose was fed to add 4 g L^-1^ in the cultures at a 96 h and b 120 h. c standard batch cultivation without feed. Cultures were prepared according to method SP4. No differences in illudin M titers were observed after feeding of glucose. Illudin M titers were derived from cell free culture supernatant.

Fig. S 3 Illudin M, pH and substrate kinetics of cultures of *O. nidiformis* fed with acetate (CH_3_CO_2_K) at different time points. Acetate (CH_3_CO_2_K) was fed to reach a concentration of 8 g L^-1^ at a 0 h, b 72 h, c 96 h, d 120 h and e served as control without feed. The data shows titers of Illudin M (blue), glucose (red), acetate (black) and the pH (green) plotted over the cultivation time. f shows the highest illudin M titers achieved in this trial. All experiments were conducted in G13.5/C7 medium. Cultures were prepared according to method SP4. Illudin M titers were derived from cell free culture supernatant.

Fig. S 4 Illudin M, pH and substrate kinetics from cultures of *O. nidiformis* fed with acetic acid. The feed was performed at 96 h to add 8 g L^-1^ of acetate. The solution of acetic acid was titrated with KOH in order to add the potassium ion and to adjust the pH to 4.2 that is the typical pH of the batch cultivation at 96 h. Curves are colored according to the colors of the labels of each axis. After the feed, the production of illudin M did not increase, glucose and acetate were no longer consumed and pH did not change. Kinetics and visual observation of the cultures indicated a potential growth inhibition. Illudin M titers were derived from cell free culture supernatant.

Fig. S 5 Illudin M, pH and substrates kinectics from cultures of *O. nidiformis* fed with acetate from two different salts (CH_3_CO_2_K; CH_3_CO_2_Na). CH_3_CO_2_K was fed to reach a concentration of a 8 g L^-1^ at 96 h and b two times feed of 4 g L^-1^ (96 h and 168 h and 168 h). CH_3_CO_2_Na was fed to reach a concentration of c 8 g L^-1^ at 96 h and d two times feed of 4 g L^-1^ (96 h and 168 h and 168 h). All cultures were additionally fed with glucose (total addition 6 g L^-1^) at 120 h. e Control experiment without feed. All cultures were prepared using G13.5/C7 medium according to method SP4. Illudin M titers were derived from cell free culture supernatant.


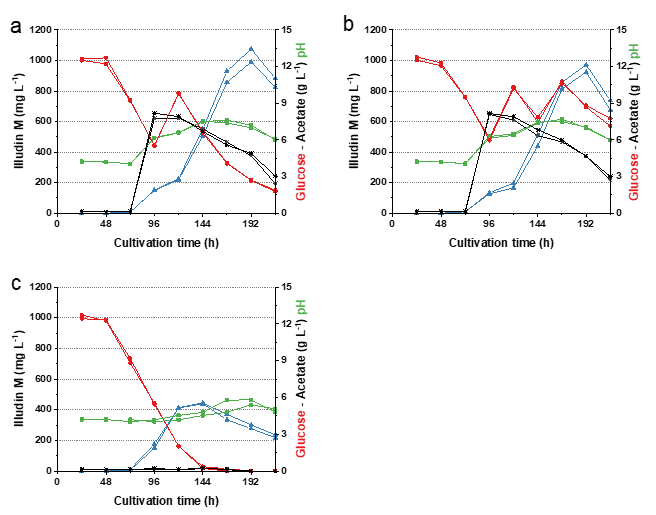


Fig. S 6 Illudin M, pH and substrate kinetics from cultures of *O. nidiformis* fed with acetate and glucose.. Cultures were fed to reach a concentration of 8 g L^-1^ acetate from CH_3_CO_2_K at 96 h and a one time feed of glucose (total addition 6 g L^-1^) at 120 h, b two feeds of glucose (total addition 6 g L^-1^) at 120 h and 168 h. c control experiment without feed. All cultures were prepared using G13.5/C7 medium according to method SP4. Illudin M titers were derived from cell free culture supernatant.


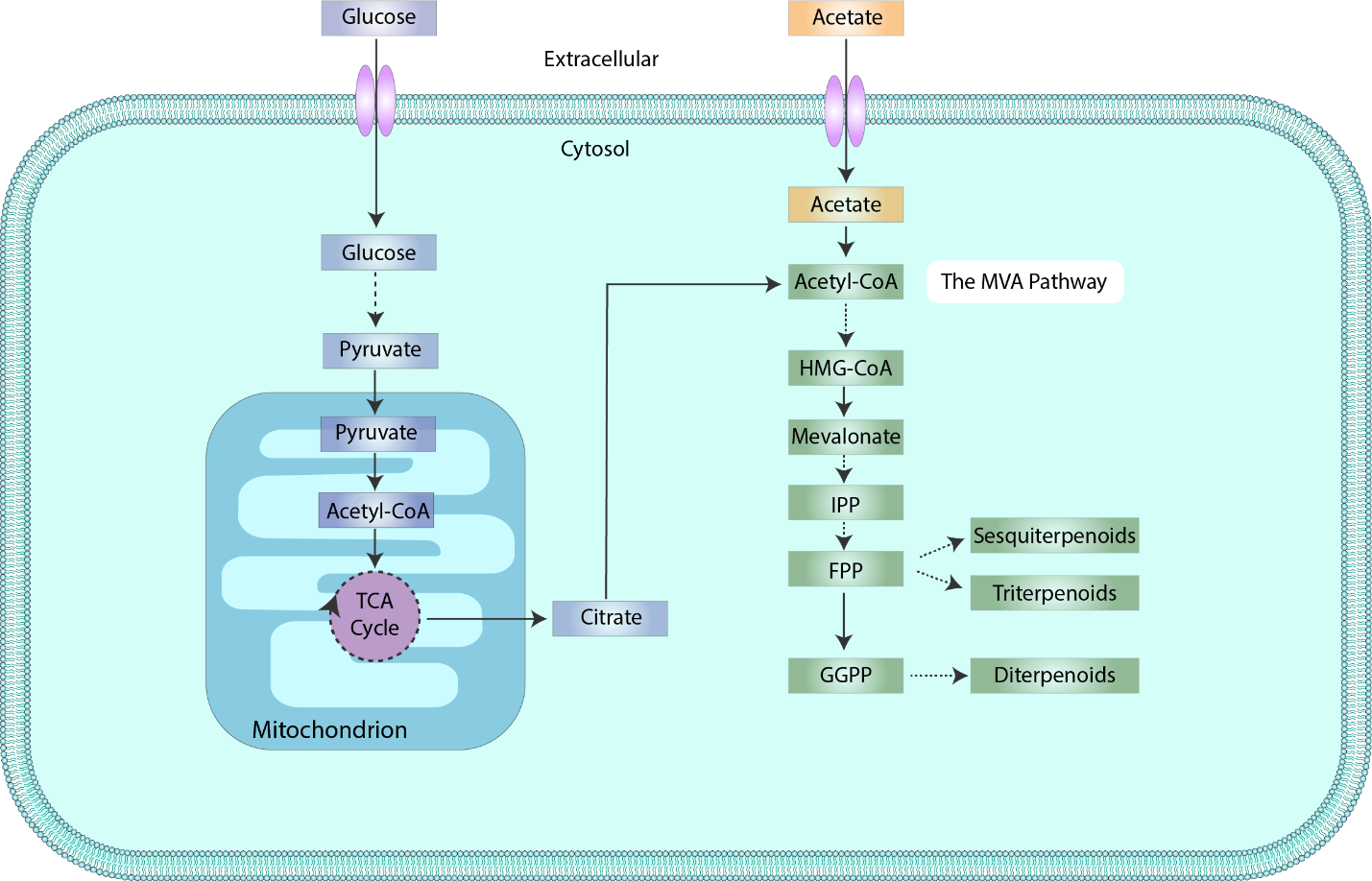


Fig. S 7 General overview of glycolysis and the mevalonate (MVA) pathway in eukaryotes. During oxidation of glucose for respiration activity pyruvate enters the mitochondrion and it is converted into acetyl-CoA for incorporation in the tricarboxylic acid cycle (TCA). One way to obtain acetyl-CoA in the cytosol is via conversion of citrate. Then, acetyl-CoA can serve as precursor for the MVA pathway which is an important anabolic pathway for the production of terpenes. Metabolites involved in the MVA pathway are shown in green boxes and metabolites involved in glycolysis are shown in purple boxes. Dashed arrows indicate multiple steps in the pathways. This illustration has been simplified and does not present complete or interconvertion reactions. FPP, farnesyl diphosphate; GGPP, geranylgeranyl-diphosphate; HMG-CoA, 3 hydroxy 3 methylglutaryl CoA; IPP, isopentenyl-diphosphate. This illustration was adapted from (1).

**1. Mullen PJ, Yu R, Longo J, Archer MC, Penn LZ. The interplay between cell signalling and the mevalonate pathway in cancer. Nat Rev Cancer. 2016;16(11):718–31.**
